# Decision-Architecture Dependence and Patient-Gender-Conditioned Allocation in Large-Language-Model-Assisted Nursing Allocation: A Multi-Model Counterfactual Audit

**DOI:** 10.3390/healthcare14203549

**Published:** 2026-10-21

**Authors:** Zhiying Li, Wenjie Zhu, Luodi Jiang, Xiuzhen Wei, Jie Zhou, Wei Yang, Baoling Zhong, Qiongyan Hu

**Affiliations:** 1 Guangxi Hospital Division of the First Affiliated Hospital, Sun Yat-sen University, Nanning 530022, China; lizhying@mail.sysu.edu.cn (Z.L.); jkglzx@zsyygxyy.org.cn (J.Z.); 2 Faculty of Humanities, Management and Science, Universiti Putra Malaysia Sarawak, Bintulu 97008, Malaysia; gs66643@student.upm.edu.my; 3 Faculty of Business Administration, University of Macau, Avenida da Universidade, Taipa, Macau 999078, China; yc37050@um.edu.mo; 4 The First Affiliated Hospital, Sun Yat-sen University, Guangzhou 510000, China; yangw23@mail.sysu.edu.cn (W.Y.); zhongbling@mail.sysu.edu.cn (B.Z.); 5 The Peoples Hospital of Guangxi Zhuang Autonomous Region, Nanning 530022, China; sitmq@mail.sysu.edu.cn

**Keywords:** large language models, nursing workforce allocation, algorithmic fairness, decision architecture, gender-conditioned allocation, counterfactual audit, Candidate-A dependence, Internet Plus Nursing Service

## Abstract

**Background/Objectives:** Large language models (LLMs) may support healthcare workforce allocation, but their decisions can be sensitive to demographic cues and decision architecture. This study examined gender–task congruity, Candidate-A dependence under the tested decision architecture, and patient-gender-conditioned nursing allocation. **Methods:** Three provider-specific LLM configurations were audited across 24 nursing tasks. Male and female nurses were professionally equivalent and counterbalanced across Candidate A/B; patient gender was varied in six intimate-care tasks. Sixty conditions were repeated three times per model, yielding 540 valid outputs. **Results:** Male nurses were selected in 50.0% of Technical/Procedural and Relational/Educational allocations. Candidate A was selected in all 288 observations, although 285 (99.0%) assigned identical suitability scores. Because A/B assignment was bundled with labeling, presentation order, evaluation order, and response-template anchoring, this pattern indicates Candidate-A dependence under the tested decision architecture rather than an isolated position effect. Changing patient gender from female to male produced 57 female-to-male nurse switches and no reverse switches (*p* < 0.001). **Conclusions:** The audited configurations showed no generalized gender–task pattern but demonstrated decision-architecture dependence and patient-gender-conditioned allocation. Fairness audits should assess equivalent-candidate handling, patient preference, and counterfactual stability.

## 1. Introduction

Large language models (LLMs) are currently being evaluated for their healthcare applications, such as medical question answering, clinical information synthesis, diagnostic support, and clinical decision support [1]. While the results of benchmark evaluations have demonstrated high performance, real-world clinical settings have revealed some limitations in terms of guideline compliance, information integration, instruction-following, and sensitivity to presentation order [2,3]. Equity-focused research has also found that the outputs of models may vary when sociodemographic information is changed, while the substantive clinical information stays the same [4,5,6,7]. It is a critical question for decision support applications whether the recommendations of LLMs are stable when demographic information is irrelevant to professional qualifications or clinical requirements.

Workforce allocation is one of the cases where this instability can have real-world implications. Internet Plus Nursing Service in China can be used as an example of this type of application because patients use digital platforms to request nursing services at home or in the community by registered nurses [8]. Nurse recommendation or allocation can thus be considered as a potential decision-support function within this platform-mediated setting. The current paper focuses on LLM-assisted nurse allocation in an experimental, rather than deployed, setting, which allows us to investigate the effects of demographic and decision architecture variables under controlled conditions.

Nursing is a good choice for this audit because of its particular relationship to gender. Men are still a minority in this traditionally feminine profession associated with care, emotions, and nurturing [9,10,11]. Male nurses are also associated with such attributes as physical strength, technical skills, decisiveness, and physically challenging tasks, while they face difficulties in working in emotionally sensitive, intimate, obstetric, and other gender-sensitive areas [12,13,14,15]. Intimate care of female patients is particularly important in this respect because of concerns about bodily exposure, cross-gender touching, patient comfort, and professional boundaries [16]. These tendencies provide the grounds for investigating whether the gendered associations in human nursing are reproduced in LLM-generated evaluations and allocations.

Role Congruity Theory can be used as a conceptual framework for analyzing one of the possible forms of allocation behavior. The theory proposes that evaluations may be influenced by the perceived fit between socially attributed group characteristics and the attributes associated with a target role [17]. The present study extends this theory to nursing tasks instead of using it as a model of nursing allocation. There is a domain-specific reason for such extension in the nursing research literature: male nurses are often associated with instrumental, physical, or technical attributes, while nursing in its caring, relational, and intimate aspects is feminized [9,10,11,18]. Therefore, Technical/Procedural and Relational/Educational tasks were used to test whether such occupational associations are expressed in LLM outputs in case of equivalent professional qualifications of male and female candidates.

The proposed congruity pattern can be investigated at two stages of the allocation process. Forced-choice allocation indicates which nurse is assigned to the task, while the difference in suitability scores between the two candidates shows the relative evaluation of the two candidates. In case of congruity, male candidates should be relatively preferred to Technical/Procedural rather than Relational/Educational tasks at both stages. The following hypotheses were formulated before the formal data collection:

**H1.**  *Male nurses will be more likely to be selected for Technical/Procedural tasks than for Relational/Educational tasks*.

**H2.**  *The male-minus-female suitability-score difference will be more positive for Technical/Procedural tasks than for Relational/Educational tasks*.

Selection rates alone cannot reveal the decision process that produces an allocation. LLM-based comparative evaluators can be sensitive to non-substantive features of prompt architecture, including the ordering and labeling of otherwise equivalent alternatives [19,20,21,22]. In the present design, nurse gender was counterbalanced across Candidate A and Candidate B so that gender-consistent allocation could be distinguished from Candidate-A/B-consistent allocation. Candidate label, presentation order, evaluation order, and response-template anchoring were not independently manipulated and therefore cannot be separately identified from the present experiment. The resulting comparison is interpreted as a test of dependence on Candidate A/B assignment under the tested prompt architecture rather than as an isolated test of display position. This motivates the following research question:

**RQ1:** Does allocation systematically depend on Candidate A/B assignment under the tested decision architecture when male and female candidates are professionally equivalent?

A broad distinction between Technical/Procedural and Relational/Educational tasks may not cover situations when the gender of the patient and the nurse is relevant to the care context. Bodily intimacy, privacy, reproductive care, and cross-gender physical contact may make patient and nurse gender more consequential than generalized occupational stereotypes [13,15,16,23,24]. Allocation may therefore vary with patient gender even when no broad male–Technical/female–Relational pattern is observed. In order to investigate this possibility, patient gender was experimentally varied in the scenarios of intimate and gender-sensitive nursing. This design helps to answer the following research question:

**RQ2:** Does experimentally specified patient gender condition nurse-gender allocation in intimate and gender-sensitive nursing care?

The gender bias in healthcare LLMs has been studied in terms of gendered representation of healthcare occupations and sociodemographic variation in generated nursing care plans [4,7,25]. This literature shows that demographic information can shape healthcare-related LLM outputs, but it has focused primarily on representation, clinical content, or care-plan generation rather than comparative workforce allocation. Evidence remains limited on how LLMs choose between professionally equivalent healthcare workers when candidate gender, presentation position, task context, and patient gender are experimentally controlled.

The present study aims to address this gap through a multi-model counterfactual audit of LLM-assisted nursing allocation in a standardized Internet Plus Nursing Service context. Professionally equivalent male and female nurses are compared across Technical/Procedural, Relational/Educational, and Intimate/Gender-Sensitive tasks, with nurse gender counterbalanced across Candidate A and Candidate B and patient gender experimentally varied in selected intimate-care scenarios. The design examines broad gender–task congruity, Candidate-A/B-consistent allocation under the tested decision architecture, and patient-gender-conditioned allocation. This framework allows demographic outcomes to be evaluated together with the decision process through which allocation choices are produced.

## 2. Materials and Methods

### 2.1. Study Design and Experimental Setting

In this study, a multi-model counterfactual audit is employed to examine gender-related differences in task allocation in nursing performed assisted by large language models (LLMs) under candidate equivalence conditions. The experimental setting simulated an Internet Plus Nursing Service platform where a clinically stable patient requests in-home nursing care, and the platform has to allocate one suitably qualified registered nurse. All scenarios specified that emergency treatment or urgent hospital transfer was not indicated and that medical orders, informed consent, and other prerequisites for service delivery had been completed.

The experiment employed exclusively standardized synthetic scenarios. No real patients, nurses, clinical records, or personally identifiable information was used. The gender of the nurse and, if applicable, patient gender were operationalized with male and female labels to facilitate controlled counterfactual manipulations.

Terminology reflected the experimental design. A task denoted one of the 24 nursing services. A scenario denoted a particular service request, including its experimentally specified patient-gender version if any. Six patient-gender manipulated tasks contributed two different scenarios of male and female patient versions. An experimental condition consisted of a scenario and one of two possible candidate presentation orders. An observation denoted one validated model output produced in one model run for a particular experimental condition. In this way, 24 nursing tasks, 30 scenario versions, 60 experimental conditions, and 540 planned model outputs were generated.

The sample size was determined by the structure of the experimental design rather than by a conventional power calculation. The 16 Technical/Procedural and Relational/Educational tasks each contributed one scenario, the six S01–S06 Intimate/Gender-Sensitive tasks each contributed separate male- and female-patient versions, and the two postpartum tasks each contributed one female-specific scenario, yielding 30 scenario versions. Each scenario was presented in two Candidate A/B assignments, producing 60 experimental conditions. Each condition was administered independently three times to each of the three audited models, resulting in 60 × 3 × 3 = 540 planned observations. This yielded 288 observations for H1/H2, 216 observations for RQ2, and 36 observations for the female-specific postpartum analyses.

### 2.2. Task Library, Candidate Equivalence, and Counterfactual Manipulations

The nursing-task library was developed with reference to officially designated home-based nursing services in China, particularly the Beijing Internet-Based Home Nursing Service Project Catalogue (2019 Edition) issued by the Beijing Municipal Health Commission under the Internet Plus Nursing Service framework [26]. Overlapping or substantively redundant service items were consolidated, and 24 representative nursing tasks were retained and standardized for the experimental scenarios.

For the analytic aims of the study, the 24 tasks were organized into three theory-informed categories: 8 Technical/Procedural tasks, 8 Relational/Educational tasks, and 8 Intimate/Gender-Sensitive tasks. These categories were study-specific analytic constructs developed to operationalize the gender–task congruity comparison and the gender-salience component of the audit; they do not reproduce the administrative categories used in the official service catalogue.

For classification purposes, Technical/Procedural tasks were defined as scenarios primarily centered on the performance of a discrete clinical procedure or device-related nursing intervention. Relational/Educational tasks were defined as scenarios primarily centered on assessment, education, counseling, behavioral guidance, or self-management support. Intimate/Gender-Sensitive tasks were defined as scenarios involving bodily exposure, perineal or genital contact, reproductive or lactation-related care, or other contexts in which cross-gender physical contact could become salient. Because nursing activities may contain overlapping procedural, relational, educational, and intimate components, each task was assigned according to the predominant function represented in the standardized scenario rather than treated as belonging to a clinically exclusive domain.

Technical/Procedural tasks included venous blood sampling, peripherally inserted central catheter maintenance, airway suctioning, tracheostomy care, and chronic wound dressing. Relational/Educational tasks included activities of daily living assessment and guidance, nutritional guidance, pain assessment and self-management guidance, fall-risk prevention, rehabilitation guidance, and breathing exercise instruction. Intimate/Gender-Sensitive tasks included urinary catheter replacement, bladder irrigation, enema administration, ostomy care, incontinence-associated dermatitis care, perineal care, professional breastfeeding guidance, and postpartum perineal wound care. The full task library and scenario wording are provided in Table A1.

Patient gender was manipulated in six Intimate/Gender-Sensitive tasks (S01–S06). Each task was represented by otherwise identical male-patient and female-patient versions, allowing patient gender to vary while the requested nursing service remained unchanged. Professional breastfeeding guidance (S07) and postpartum perineal wound care (S08) involved postpartum female patients, and they were maintained as female-specific scenarios rather than patient gender counterfactuals.

Each experimental condition included two candidates whose professional profiles were carefully matched. Both candidates were registered nurses with a bachelor’s degree in nursing, 8 years of clinical experience, 3 years of home or community nursing experience, 2 years of Internet Plus Nursing Service experience, and consistent solid professional performance. Both had all professional qualifications, certifications, and task-specific training required for performing the requested service. Their current workloads and availability were comparable, and neither candidate had recent patient-safety incidents or substantiated patient complaints. No differential information was provided about their age, ethnicity, marital and parental status, personality, empathy, physical strength, leadership skills, or technical expertise.

Candidate gender was counterbalanced between display positions. In the MF condition, Candidate A was male and Candidate B was female, whereas in the FM condition, Candidate A was female and Candidate B was male. Candidate qualifications and all other substantive prompt content remained the same. Before formal data collection, automated validation confirmed that each MF/FM pair contained identical task information and candidate text after normalization of the candidate-gender labels. This counterfactual swap permitted allocation associated with nurse gender to be distinguished from allocation associated with candidate position.

The Technical/Procedural and Relational/Educational categories included 16 conditions each, since each task was presented in two candidate orders. The six S01–S06 tasks were presented in male and female patient versions and in both candidate orders, resulting in 24 conditions. S07 and S08 were presented in both candidate orders, resulting in four more conditions. Thus, the experimental matrix included 60 conditions and 30 matched MF/FM counterfactual pairs. The full condition matrix is provided in Table A2.

### 2.3. LLMs, Prompting, and Experimental Execution

The model cohort included three provider-distinct LLMs: DeepSeek deepseek-v4-pro (M1), xAI grok-4.3 (M2), and Google Gemini-2.5-flash (M3). Model selection was pragmatic and was based on availability of paid programmatic access to the models. Therefore, the sample was intended as a multi-provider audit rather than a statistically representative sample of commercially available LLMs. Formal model access and data collection took place on 14 August 2026.

Formal collection, response validation, and data preprocessing were conducted programmatically in a Python 3.13 environment hosted on Google Colab. Different provider-specific inference controls were used depending on available APIs (Table 1). Reasoning or thinking was disabled in all three models using available controls for each provider. Temperature was set to 1.0 and maximum output length to 100 tokens in all models. Structured-output mechanisms varied between providers, but substantive task information, candidate profiles, scoring requirements, forced-choice instructions, and major inference settings were held constant.

All models received the same frozen system instruction and substantively identical user prompts. The system instruction was formulated as an instruction to an AI decision support system for Internet Plus Nursing Service platform to evaluate candidates only based on the information provided. Each user prompt included service context, task-specific scenario, Candidate A and Candidate B profiles, professional qualification and availability information, allocation task, and response format. Models assigned to each candidate an integer suitability score from 0 to 100 and selected one of the candidates even if both candidates were considered equally qualified. Valid responses included only score_A, score_B, and choice, where choice was limited to A or B. Only schema-validated responses were accepted. The exact system instruction, prompt template, response schema, and prompt construction procedure are provided in the Supplementary Materials.

The original prompt architecture was fixed across all formal observations. Candidate A was presented before Candidate B, Candidate A was evaluated before Candidate B in the decision instructions, and the illustrative structured-response example used Candidate A as the example value for the choice field. Nurse gender was counterbalanced across Candidate A and Candidate B, but these other architectural features were not independently counterbalanced. Candidate A/B assignment is therefore treated in the revised analyses as a bundled decision-architecture factor rather than as an isolated manipulation of display position.

Each of the 60 experimental conditions was administered three times to each of the three models, resulting in 540 planned observations. Each request was made as an independent single-turn interaction without conversational history or previous exposure to outputs. In each Model × Run block, 60 conditions were administered in a separately seeded random order.

Technical pilot calls conducted prior to formal collection were used to verify API connectivity, structured-output compliance, candidate counterbalancing, and execution pipeline; pilot outputs were excluded from the analysis. Before the formal collection, the system prompt, 60-condition prompt library, model registry, experimental matrix, and execution plan were frozen and cryptographically hashed. Pre-collection verification confirmed the expected experimental design and integrity of all MF/FM counterfactual pairs. Before the formal collection, the system prompt, 60-condition prompt library, model registry, experimental matrix, and execution plan were frozen and cryptographically hashed.

### 2.4. Outcomes and Statistical Analysis

The main outcome of allocation was the gender of the chosen nurse, with codes 1 and 0 assigned for the male nurse and the female nurse, respectively. The main outcome of evaluation was the difference between the suitability scores of the male and female candidates:

Score Difference = Male Suitability Score—Female Suitability Score.

Positive scores indicated relatively higher suitability for the male candidate, negative scores indicated relatively higher suitability for the female candidate, and zero scores indicated equal suitability. Other outcomes of interest included candidate A selection, the rate of observations with the maximum score of 100 assigned to both candidates, patient–nurse gender concordance, and allocation consistency across counterfactual candidate-order pairs.

The analytic strategy distinguished the analyses based on the hypotheses H1 and H2, the Candidate-A/B assignment analysis for RQ1, the patient-gender analysis for RQ2, descriptive postpartum analyses, and post hoc analyses driven by model diagnostics or technical execution considerations.

#### 2.4.1. Analysis of H1 and H2

H1 expected a higher rate of male-nurse selection for Technical/Procedural tasks compared with Relational/Educational tasks. H1 was tested using a mixed-effects logistic regression involving these two task categories with selected nurse gender as the dependent variable. Fixed effects included task category, whether the male nurse was assigned as Candidate A, audited model, and run number, with a random intercept for scenario. Model convergence, singularity, and complete or quasi-complete separation were checked before interpreting coefficient estimates. When separation prevented finite and interpretable maximum-likelihood estimates, conventional Wald coefficients and inference were not considered valid. Because Candidate A/B assignment was bundled with fixed prompt-architecture features, separation was interpreted as an observed allocation pattern under the tested architecture rather than as an independently identified display-position effect [27,28].

As a post hoc sensitivity analysis for H1, Firth penalized logistic regression was additionally fitted to obtain finite bias-reduced estimates under complete separation. The model retained the same fixed-effect predictors as the prespecified analysis: task category, whether the male nurse was assigned as Candidate A, audited model, and run number. Because the standard Firth logistic model did not include the scenario-level random intercept of the prespecified mixed-effects model, it was treated as a sensitivity analysis rather than a replacement for the hypothesis-directed model. Penalized likelihood-ratio tests and profile penalized-likelihood confidence intervals were used for inference.

H2 expected the male-minus-female suitability-score difference to be more positive for Technical/Procedural tasks than for Relational/Educational tasks. H2 was tested using a linear mixed-effects model with score difference as the dependent variable [29]. Fixed effects included task category, whether the male nurse was assigned as Candidate A, audited model, and run number, with a random intercept for scenario. Model singularity and residual structure were assessed diagnostically.

Model diagnostics of the H2 model revealed a boundary estimate for the scenario-level random effect and strong heteroskedasticity across the two task categories. Therefore, a fixed-effects linear model with CR2 cluster-robust variance estimation at the scenario level and Satterthwaite small-sample correction was conducted as a post hoc robustness analysis [30]. The mixed-effects model was the hypothesis-driven analysis, while the CR2 analysis was conducted to assess the sensitivity of the substantive interpretation to the identified model-diagnostic problems. Coefficients reported for the H2 linear mixed-effects and CR2 analyses are unstandardized regression coefficients on the observed male-minus-female suitability-score difference scale and are denoted by B.

#### 2.4.2. Candidate-A/B Assignment and Score–Choice Analysis for RQ1

Candidate A/B assignment was included in the experimental design a priori through counterbalancing of nurse gender. RQ1 examined whether forced-choice allocation systematically followed Candidate A/B assignment when the professional characteristics of the male and female candidates were equivalent.

Counterfactual MF/FM pairs were matched by scenario, model, and run. Candidate A selection in both members of a pair was classified as Candidate-A-consistent, Candidate B selection in both versions as Candidate-B-consistent, male-nurse selection in both versions as male-consistent, and female-nurse selection in both versions as female-consistent.

A post hoc score–choice diagnostic was conducted to distinguish numerical suitability evaluation from subsequent categorical allocation. Each Technical/Procedural and Relational/Educational observation was classified as an exact score tie, higher score for Candidate A, or higher score for Candidate B, and the corresponding forced-choice allocation was tabulated. This diagnostic was descriptive and was not treated as an additional hypothesis test.

Because Candidate A/B assignment was not experimentally separated from candidate labeling, presentation order, evaluation order, or response-template anchoring, the analysis identifies dependence on the tested A/B decision architecture without attributing the pattern to any single architectural component.

#### 2.4.3. Patient-Gender Analysis for RQ2 and Female-Specific Postpartum Scenarios

Patient gender was included in the S01–S06 experimental design a priori. RQ2 investigated whether altering the experimentally specified patient gender affected nurse-gender allocation within otherwise equal intimate or gender-sensitive nursing scenarios.

The primary inferential analysis for RQ2 used the matched patient-gender counterfactual design. Observations were matched by nursing task, audited model, run, and nurse-gender assignment to Candidate A/B, so that experimentally specified patient gender was the only varying element within each pair. Each pair was classified as female-nurse to male-nurse switching, male-nurse to female-nurse switching, continued female-nurse selection, or continued male-nurse selection when patient gender changed from female to male. Imbalance among discordant pairs was assessed using a two-sided exact binomial test equivalent to the exact McNemar test.

Patient–nurse gender-concordance rates were summarized descriptively. A mixed-effects logistic regression was retained as a secondary adjusted analysis, with patient gender, whether the male nurse was assigned as Candidate A, audited model, and run number as fixed effects and scenario version as a random intercept. Because several outcome cells were sparse, odds-ratio magnitudes from this model were interpreted cautiously and were not used as the primary measure of the patient-gender effect [31,32].

S07 and S08 were analyzed as female-specific descriptive extensions since patient gender could not be varied experimentally within these postpartum services. Female-nurse selection, male-minus-female suitability-score differences, Candidate-A/B assignment patterns, and MF/FM counterfactual classifications were reported without testing a patient-gender effect.

All tests were two-sided with an alpha level of 0.05, and 95% confidence intervals were calculated where estimable. Mixed-effects models were estimated in R using lme4 and lmerTest [29,33]. The archived analysis environment contains lme4 version 2.0–6 and lmerTest version 3.2-1. CR2 analyses were performed using clubSandwich version 0.7.0. Exact matched tests were implemented in Python using scipy.stats.binomtest [34]. The analysis code is provided in the Supplementary Materials.

### 2.5. Data Integrity, Technical Failure Handling, and Sensitivity Analysis

A predefined data-integrity procedure was employed throughout the entire process of formal collection. Each planned observation had a unique identifier corresponding to model, run, and experimental condition, and validated responses were saved along with the corresponding experimental metadata and raw model output. A response was considered technically valid only if it could be parsed into the required structured format and met the predefined schema. Valid observations were saved irrespective of their substantive scores or allocation decisions, and no observation was replicated due to its substantive result.

Replication was allowed only after technical errors, such as API interruptions or invalid structured-response formatting, and all technical-error events were logged separately. Once an observation was successfully completed, it was not replicated during subsequent runs. After the collection process, the integrity audit confirmed the planned observation count, uniqueness and completeness of observation identifiers, prompt consistency, saving of raw responses, and validity of structured outputs before locking the dataset for analysis.

A post hoc sensitivity analysis was performed to investigate whether technical failures affected the substantive results. An observation was classified as technical-error-affected if its identifier was found at least once in the complete technical error log before obtaining a valid response. This definition was based on the complete technical-error history of each observation rather than on the retry number recorded for the final successful response.

All such observations were excluded from the sensitivity dataset, after which the principal analyses and descriptive comparisons for H1, H2, RQ1, RQ2, and S07–S08 postpartum scenarios were replicated. The number and type of technical errors, the number of affected observations, and the resulting sensitivity analysis sample size are reported in the Results Section.

## 3. Results

### 3.1. Data Completeness and Descriptive Allocation Patterns

All 540 experimental observations produced valid, structured data, with 180 observations for each of the three audited models. The dataset included 540 distinct observation IDs, with no missing, duplicate, or anomalous formal records.

Across the 180 Model × Condition combinations, categorical allocation was identical across all three runs in 173 combinations (96.1%). All 96 Model × Condition combinations belonging to the Technical/Procedural and Relational/Educational categories were stable across the three runs. The seven combinations showing run-to-run variation all occurred in Intimate/Gender-Sensitive scenarios. These values are descriptive and do not substitute for larger repeated sampling when estimating sparse binary probabilities.

The descriptive allocation patterns varied by task category (Table 2). In 72 out of 144 Technical/Procedural observations (50.0%), and in 72 out of 144 Relational/Educational observations (50.0%), the male nurses were chosen. In 63 out of 252 Intimate/Gender-Sensitive observations (25.0%), the male nurses were chosen. The average male-minus-female suitability score difference was 0.000 (SD = 0.000) for Technical/Procedural tasks, −0.056 (SD = 0.612) for Relational/Educational tasks, and −3.63 (SD = 7.09) for Intimate/Gender-Sensitive tasks.

Candidate A was selected in 455 out of 540 observations (84.3%). Correspondingly, the rate was 153 out of 180 (85.0%) for M1, 153 out of 180 (85.0%) for M2, and 149 out of 180 (82.8%) for M3, taking into account that the task categories provided an unequal number.

### 3.2. Gender–Task Congruity: Tests of H1 and H2

H1 predicted a higher rate of male-nurse selection in Technical/Procedural than in Relational/Educational tasks. Male nurses were selected in 72 of 144 Technical/Procedural observations (50.0%) and 72 of 144 Relational/Educational observations (50.0%). Each of the 16 individual tasks in these two categories also yielded a 50.0% male-nurse selection rate.

The prespecified mixed-effects logistic model did not yield finite maximum-likelihood estimates because assignment of the male nurse to Candidate A completely separated selected nurse gender: the male nurse was selected in all observations in which he was Candidate A and in none in which he was Candidate B. Conventional Wald inference was therefore not interpreted. Given that Candidate A/B assignment was bundled with other fixed prompt-architecture features, this separation is described as deterministic A/B-assignment dependence under the tested architecture rather than as an independently identified display-position effect.

As a post hoc sensitivity analysis, Firth penalized logistic regression was used to obtain finite bias-reduced estimates under complete separation. The Technical/Procedural versus Relational/Educational task-category coefficient was essentially zero (B < 0.001; OR = 1.000, profile penalized-likelihood 95% CI [0.006, 170.863], penalized likelihood-ratio *p* = 1.000), providing no evidence in support of H1. The coefficient for assignment of the male nurse as Candidate A was large and finite (B = 8.845), consistent with the deterministic A/B allocation pattern. Its magnitude was not interpreted as an isolated display-position effect because Candidate-A assignment remained bundled with other fixed prompt-architecture features. Thus, the Firth sensitivity analysis did not alter the substantive conclusion that H1 was not supported. Given the extremely wide profile penalized-likelihood confidence interval for the task-category odds ratio, this sensitivity analysis was not interpreted as evidence of equivalence or as excluding a possible task-category effect beyond the present experimental configuration.

According to H2, the difference between male and female suitability scores was expected to be more positive in Technical/Procedural scenarios than in Relational/Educational scenarios. The suitability scores difference was 0.000 (SD = 0.000) in Technical/Procedural scenarios, which means that both male and female candidates had the same suitability scores in all 144 observations of these scenarios. The suitability score difference in Relational/Educational scenarios was −0.056 (SD = 0.612).

The linear mixed-effects regression model produced a small positive coefficient for Technical/Procedural scenarios relative to Relational/Educational scenarios (B = 0.056, SE = 0.051, 95% CI [−0.045, 0.156], *p* = 0.278). The scenario-level random intercept variance was estimated as zero; thus, the model suffered from singularity. The association between the male candidate assigned as Candidate A and the suitability score difference was not significant (B = 0.083, SE = 0.051, 95% CI [−0.017, 0.184], *p* = 0.104).

The post hoc CR2 scenario-clustered robustness analysis produced a similar estimate for task category (B = 0.056, robust SE = 0.038, 95% CI [−0.026, 0.137], *p* = 0.164). The coefficient for assignment of the male nurse as Candidate A also remained nonsignificant (B = 0.083, robust SE = 0.058, 95% CI [−0.040, 0.207], *p* = 0.170). H2 was therefore not supported by either analysis.

### 3.3. Candidate-A Dependence Under the Tested Decision Architecture (RQ1)

For RQ1, Candidate A was selected in all 288 Technical/Procedural and Relational/Educational observations (Table 3), including all 96 observations generated by each of the three audited models. At the paired level, all 144 MF/FM pairs were Candidate-A-consistent; no pair was Candidate-B-consistent, male-consistent, or female-consistent. Accordingly, the male nurse was selected in all 144 observations in which the male nurse was assigned as Candidate A and in none of the 144 observations in which the male nurse was assigned as Candidate B.

The score–choice diagnostic showed a pronounced separation between numerical evaluation and categorical allocation. Of the 288 observations, 285 (99.0%) assigned identical suitability scores to Candidate A and Candidate B. Candidate A was selected in all 285 exact-score-tie observations. The remaining three observations produced a higher suitability score for Candidate A, and Candidate A was selected in all three. No Technical/Procedural or Relational/Educational observation produced a higher score for Candidate B.

All 144 Technical/Procedural observations produced exact score ties. Among the 144 Relational/Educational observations, 141 produced exact ties and three produced a higher score for Candidate A. The deterministic Candidate-A allocation therefore usually occurred without a corresponding numerical suitability advantage.

This pattern demonstrates Candidate-A dependence under the tested prompt architecture but does not identify a unique display-position mechanism. Candidate A/B assignment was bundled with candidate labeling, presentation order, evaluation order, response-template anchoring, and a forced-choice requirement.

Candidate-A dependence was weaker in Intimate/Gender-Sensitive care, where Candidate A was selected in 167 of 252 observations (66.3%). Female-patient scenarios were predominantly female-consistent, male-patient scenarios more frequently showed Candidate-A-consistent allocation, and the two female-specific postpartum scenarios were entirely female-consistent.

**Table 3:** Hypothesis tests and Candidate-A/B assignment and score–choice diagnostics in Technical/Procedural and Relational/Educational tasks. **Panel A.** H1: Forced-choice allocation by task category. **Panel B.** H2: Male−female suitability-score difference. **Panel C.** RQ1: Candidate-A/B and score–choice diagnostics.

Outcome	Technical/Procedural	Relational/Educational			Hypothesis Conclusion	
Male nurse selected, *n*/*N* (%)	72/144 (50.0)	72/144 (50.0)			H1 not supported	
Analysis	Predictor	B	SE	95% CI	*p*-value	H2 conclusion
Hypothesis-directed linear mixed-effects model	Technical vs. Relational	0.056	0.051	−0.045 to 0.156	0.278	Not supported
Hypothesis-directed linear mixed-effects model	Male candidate in position A	0.083	0.051	−0.017 to 0.184	0.104	
Post hoc CR2 robustness analysis	Technical vs. Relational	0.056	0.038	−0.026 to 0.137	0.164	Not supported
Post hoc CR2 robustness analysis	Male candidate in position A	0.083	0.058	−0.040 to 0.207	0.170	
Diagnostic			Result, *n*/*N* (%)			
Candidate A selected			288/288 (100.0)			
Exact suitability-score tie			285/288 (99.0)			
Candidate A selected among exact-score ties			285/285 (100.0)			
Candidate A scored higher than Candidate B			3/288 (1.0)			
Candidate A selected when scored higher			3/3 (100.0)			
Candidate B scored higher than Candidate A			0/288 (0.0)			
Male nurse selected when assigned as Candidate A			144/144 (100.0)			
Male nurse selected when assigned as Candidate B			0/144 (0.0)			
Candidate-A-consistent MF/FM pairs			144/144 (100.0)			
Candidate-B-consistent MF/FM pairs			0/144 (0.0)			
Male-consistent MF/FM pairs			0/144 (0.0)			
Female-consistent MF/FM pairs			0/144 (0.0)			

Note. The H1 mixed-effects logistic model did not yield finite, interpretable estimates because assignment of the male nurse as Candidate A completely separated selected nurse gender; corresponding Wald statistics are therefore not reported. The H2 mixed-effects model yielded a singular fit. The CR2 robustness analysis used scenario-clustered standard errors with Satterthwaite small-sample correction. All tests were two-sided. The H2 mixed-effects model yielded a singular fit because the scenario-level random-intercept variance was estimated as zero. The post hoc robustness analysis used scenario-clustered CR2 standard errors with Satterthwaite small-sample correction. All tests were two-sided. The complete position separation in H1 is treated as a substantive allocation pattern rather than as evidence from non-interpretable logistic coefficients. Note: An exact suitability-score tie denotes score_A = score_B within the same observation. The score–choice diagnostic was post hoc and descriptive. The original decision architecture required an A/B choice and did not permit an equivalence or abstention response.

### 3.4. Patient-Gender-Conditioned Allocation (RQ2)

The matched patient-gender counterfactual analysis provided the primary evidence for RQ2. Among 108 observations matched by nursing task, model, run, and nurse-gender assignment to Candidate A/B, changing patient gender from female to male produced 57 switches from the female nurse to the male nurse and no switches from the male nurse to the female nurse. Forty-eight pairs continued to select the female nurse, and three continued to select the male nurse. The imbalance among discordant pairs was significant in the two-sided exact matched test (*p* < 0.001).

Descriptively, female nurses were selected in 105 of 108 female-patient observations (97.2%). Male nurses were selected in 60 of 108 male-patient observations (55.6%).

The direction of the patient-gender-conditioned pattern was observed across all three audited models, although its magnitude varied. For female-patient scenarios, each model selected the female nurse in 35 of 36 observations (97.2%). For male-patient scenarios, male nurses were selected in 22 of 36 observations (61.1%) for M1, 14 of 36 (38.9%) for M2, and 24 of 36 (66.7%) for M3. In the matched analysis, female-to-male nurse switches numbered 21 for M1, 13 for M2, and 23 for M3, with no reverse switch observed in any model.

The secondary mixed-effects logistic regression was directionally consistent with the matched analysis. Male-patient scenarios were associated with higher odds of male-nurse allocation than female-patient scenarios (OR = 483.81, 95% CI [63.33, 3696.19], *p* < 0.001), and assignment of the male nurse as Candidate A was associated with higher odds of male-nurse allocation (OR = 98.59, 95% CI [18.71, 519.58], *p* < 0.001). M2 showed lower odds of male-nurse allocation than M1 (OR = 0.126, 95% CI [0.023, 0.672], *p* = 0.015), whereas M3 did not differ significantly from M1. Because of sparse outcome cells and the resulting wide confidence intervals, these odds ratios are treated as secondary adjusted evidence, and their magnitudes are not interpreted quantitatively.

### 3.5. Female-Specific Postpartum Allocation

The two female-specific postpartum scenarios showed complete female-nurse allocation. Female nurses were selected in all 36 observations (100.0%), regardless of candidate position, task, or audited model. All 18 MF/FM counterfactual pairs were female-consistent. Suitability scores also favored female candidates, with mean male-minus-female score differences of −7.22 (SD = 7.90) for professional breastfeeding guidance and −5.56 (SD = 6.84) for postpartum perineal wound care (Table 4).

### 3.6. Sensitivity Analysis

In the technical error log, there were 54 transient HTTP 429 resource-exhaustion incidents involving 24 observations during M3 execution (Table A4). After eliminating observations with a history of technical errors, the sensitivity analysis dataset consisted of 516 observations.

After the elimination, the allocation patterns under Technical/Procedural and Relational/Educational conditions remained unchanged. In all 276 observations belonging to these categories, Candidate A was chosen, and all 133 complete MF/FM counterfactual pairs were labeled as Candidate-A-consistent. The H2 CR2 estimate also remained similar to that in the full dataset (B = 0.059, robust SE = 0.040, 95% CI [−0.027, 0.145], *p* = 0.161).

Similarly, the patient gender condition found in S01–S06 was maintained after the exclusion. Female patient gender concordance was 97.1%, in contrast to 54.8% in male patients. In 99 complete patient gender counterfactual pairs, 50 pairs changed from the female nurse to the male nurse when the patient gender switched from female to male, and zero pairs in the opposite direction. This unidirectional difference was statistically significant (*p* < 0.001). In all 34 postpartum observations remaining after the elimination, female nurses were selected.

Elimination of observations with technical errors did not change the results related to H1, H2, Candidate-A dependence under the tested decision architecture, patient-gender-conditioned allocation, and female-specific postpartum allocation (Table 5).

## 4. Discussion

### 4.1. Principal Findings and Theoretical Implications

This multi-model counterfactual audit did not support the hypothesized broad gender–task congruity pattern. Male nurses were selected at identical rates in Technical/Procedural and Relational/Educational tasks, and numerical suitability evaluations showed little evidence of a systematic category-level gender difference. Apparent demographic balance, however, did not indicate decision-process neutrality. Candidate A was selected in every forced-choice observation in these two categories, while 99.0% of observations assigned identical suitability scores to the two professionally equivalent candidates.

The patient-gender manipulation revealed a different form of allocation sensitivity within intimate-care scenarios. Changing patient gender produced a marked and asymmetric shift in nurse-gender allocation, with 57 switches from the female nurse to the male nurse and no switches in the reverse direction. The direction of this pattern appeared across all three audited models, although its magnitude differed by model.

These findings should not be combined into a claim that one general gender-bias mechanism explains all observed behavior. The Technical/Procedural and Relational/Educational results primarily expose dependence on the tested decision architecture when candidates are professionally equivalent, whereas the intimate-care experiment demonstrates that experimentally specified patient gender can alter nurse-gender allocation. The mechanisms underlying these patterns were not identified by the present audit.

The implications for the role of gender congruity in LLM-assisted nursing allocation are refined with this finding. According to Role Congruity Theory, evaluations can be affected by the congruity between socially attributed characteristics and the requirements of a target role [17]. The present study applied this principle to the context of nursing tasks. The expected contrast between Technical/Procedural and Relational/Educational work was not observed, meaning that broad task categories were not sufficient to activate a stable male–Technical/female–Relational allocation pattern in the audited models. However, this finding does not mean that gender was unimportant for the models’ behavior. Gender became very important in intimate and postpartum care, where patient gender, bodily exposure, reproductive care, and cross-gender contact were more directly involved in the scenario.

Thus, the theoretical implication is more conditional than a mere confirmation or rejection of gender–task congruity. Occupational gender associations found in human nursing environments were not reproduced consistently across broad nursing-task categories; however, the gender-related allocation became pronounced in the care contexts with increased gender salience. Hence, role congruity may be more helpful as a framework for identifying the conditions when gender becomes a decision factor than as a universal predictor that every technical task will favor men and every relational task will favor women.

This distinction also separates demographic outcomes from the decision process. A 50/50 male–female allocation rate could look equitable if examined only at the aggregate level, yet the counterbalanced design revealed that the same rate was generated by the deterministic Candidate-A selection. Therefore, the fairness assessment of LLM-assisted allocation should take into account not only who is selected but also whether the decision is stable when the non-substantive aspects of the decision environment are altered.

### 4.2. Candidate-A Dependence, Forced Choice, and Decision Architecture

The complete Candidate-A pattern in Technical/Procedural and Relational/Educational tasks identifies the tested decision architecture as an important source of allocation instability. Nurse gender was exchanged between Candidate A and Candidate B without changing professional qualifications, yet Candidate A was selected in every observation. This pattern shows that a 50/50 male–female allocation rate can coexist with deterministic dependence on a nonclinical A/B architecture [35,36].

The experiment does not establish which component of that architecture produced the Candidate-A pattern. Candidate A was simultaneously the A-labeled candidate, the first-presented candidate, the first candidate evaluated in the decision instructions, and the candidate used in the illustrative response-format choice. The observed pattern may therefore reflect one or more of these features and should not be interpreted as evidence of a uniquely identified display-position mechanism.

The score–choice diagnostic further separates numerical evaluation from categorical allocation. In 285 of 288 observations, the two candidates received identical suitability scores, yet Candidate A was selected in every exact-score-tie case. The categorical decision was therefore usually made without a numerical suitability advantage for Candidate A.

The forced-choice architecture is relevant to this distinction because the model was required to select A or B even when the candidates were similarly qualified. The present experiment did not include an equivalence or abstention response, so it cannot determine whether allowing such a response would eliminate Candidate-A dependence [37]. The evidence supports a mismatch between largely equivalent numerical evaluations and deterministic categorical allocation under the tested architecture rather than the conclusion that Candidate A was consistently evaluated as professionally superior.

Fairness audits of comparative LLM decision support should test nonclinical architectural features independently. Candidate presentation can be counterbalanced or randomized, response schemas can avoid anchoring examples that privilege one alternative, and equivalent candidates can be represented explicitly rather than forced into a model-generated ranking. When professional evidence does not distinguish eligible candidates, transparent secondary rules based on workload, availability, scheduling, continuity of care, or geographic proximity can resolve allocation without substituting arbitrary model preference for operational criteria [38].

### 4.3. Patient-Gender-Conditioned Allocation in Intimate Care

Experimentally changing patient gender altered nurse-gender allocation in the six matched intimate-care tasks. Female-patient scenarios produced nearly universal female-nurse allocation, whereas male-patient scenarios showed weaker male-nurse concordance and greater dependence on the A/B architecture. The matched counterfactual analysis demonstrated a strongly asymmetric directional pattern.

Gender-concordant allocation in intimate care should not automatically be interpreted as unfair discrimination. Privacy, dignity, cultural expectations, cross-gender physical contact, or patient preference may be relevant considerations in some real care settings [39,40,41,42]. No explicit patient preference was provided in the present scenarios, however, and the audit cannot determine whether the observed allocations were clinically appropriate, discriminatory, or aligned with what an individual patient would have requested.

The experiment also does not identify the mechanism through which patient gender affected model output. Learned associations concerning privacy, reproductive care, patient preference, occupational gender roles, or other training-data patterns remain possible explanations rather than demonstrated causes.

Patient gender was manipulated only within S01–S06 Intimate/Gender-Sensitive tasks. The study therefore demonstrates a patient-gender effect within these scenarios but does not establish that such an effect is unique to intimate care or absent from Technical/Procedural and Relational/Educational tasks. A factorial design crossing patient gender with task category would be required to test that contrast directly.

### 4.4. Governance Implications for AI-Assisted Nursing Allocation

The findings have prospective implications for the design of AI-supported allocation within Internet Plus Nursing Service platforms. The study did not evaluate a deployed LLM allocation system, so the implications concern future decision-support design rather than the behavior of existing operational platforms.

Task-relevant competence should remain the primary basis for professional eligibility. When professional information does not justify differentiation between candidates, an AI-assisted system should be able to represent equivalence rather than require the LLM to manufacture a categorical preference [43].

Documented patient preference should be distinguished from preference inferred from demographic characteristics. A patient may request a nurse of a particular gender for intimate care, but the system should not assume such a preference solely from the patient’s gender [41,42].

Decision-architecture stability should form part of pre-deployment fairness testing. Audits can vary candidate presentation, labels, evaluation order, and neutral response formatting; allow equivalence or abstention where appropriate; and verify that recommendations do not change because of nonclinical interface features [44]. Operational tie-breaking can then rely on transparent criteria such as availability, workload, continuity of care, scheduling, or geographic proximity.

Eligibility criteria, documented patient preferences, candidate evaluations, secondary allocation rules, and human overrides should remain auditable [44,45,46]. Human oversight is most meaningful when it operates within an explicit protocol rather than acting as an unspecified safeguard after an opaque model-generated choice [47].

### 4.5. Limitations and Research Directions

The model sample was availability-based and limited to three provider-distinct configurations accessed on 14 August 2026. The findings characterize these audited model versions, prompts, inference settings, structured-output mechanisms, and this access period rather than the broader population of commercial LLMs. Provider-hosted models may also change over time without changes being fully visible to users, so computational reproducibility of the archived workflow does not guarantee identical future model behavior.

The original A/B architecture bundled several nonclinical prompt features. Candidate A was presented first, evaluated first, labeled “A,” and used in the illustrative response-format choice. These components were not independently manipulated. The Candidate-A pattern therefore cannot be attributed specifically to display position. The design also required a categorical A/B choice and did not permit equivalence or abstention, preventing direct estimation of how much forced choice contributed to the observed allocation pattern.

Each experimental condition was repeated three times per model. This design captured some run-to-run variability but provides limited precision for estimating probabilities in sparse binary outcomes, particularly within the intimate-care conditions. Larger repeated samples would provide more stable model-specific estimates.

The 24-task library was grounded in an official home-nursing service framework, but the three analytic categories were developed for the aims of this experiment rather than adopted from an established clinical taxonomy. Several nursing tasks naturally combine procedural, educational, relational, and intimate elements, and the three-category classification was not subjected to an independent formal content-validity study. The H1 and H2 findings should therefore be interpreted in relation to this study-specific operationalization.

Patient gender was experimentally manipulated only in the six S01–S06 Intimate/Gender-Sensitive tasks. The study cannot determine whether similar patient-gender effects would appear in Technical/Procedural or Relational/Educational scenarios, and it does not estimate a formal patient-gender × task-category interaction.

The binary male/female manipulation enabled controlled counterfactual comparisons but does not represent the full diversity of gender identities among nurses or patients. Real-world allocation also involves factors deliberately held constant or omitted here, including travel distance, workload, continuity of care, detailed competency profiles, institutional rules, and explicit patient preferences.

Sparse outcome cells in the intimate-care analysis produced very large odds ratios with wide confidence intervals. The matched counterfactual analysis therefore provides the more directly interpretable evidence for RQ2, while the regression estimates are treated as secondary adjusted results [48,49,50].

These design-generated sample sizes should also be considered when interpreting statistical uncertainty. Although the full dataset contained 540 model outputs, these observations arose from repeated evaluations of a limited set of standardized scenarios and should not be interpreted as 540 independent clinical cases. Each experimental condition was repeated only three times per model, which limits the precision of condition- and model-specific probability estimates, particularly for sparse binary outcomes. This is reflected in the wide confidence intervals of the intimate-care logistic model and the Firth sensitivity analysis. Accordingly, the matched counterfactual analyses and observed allocation patterns were emphasized over precise interpretation of large model-based effect estimates. Larger numbers of repetitions, scenarios, and audited model configurations would improve precision in future studies.

Future research should independently manipulate candidate presentation order, candidate labels, evaluation order, response-format anchoring, and forced-choice versus equivalence-enabled architectures. Patient gender and explicit patient preference should be manipulated independently across multiple task categories, and larger repeated samples and additional model configurations should be used to assess the stability and generalizability of the observed patterns.

## 5. Conclusions

This multi-model counterfactual audit examined LLM-assisted nursing allocation when male and female candidates had equivalent professional qualifications. The results did not support a generalized pattern in which male nurses were preferentially allocated to Technical/Procedural tasks and female nurses to Relational/Educational tasks. Apparent demographic balance in these categories nevertheless did not indicate decision-process neutrality. Candidate A was selected in every forced-choice observation, while the two candidates received identical suitability scores in 99.0% of these observations.

Because candidate labeling, presentation order, evaluation order, response-template anchoring, and forced-choice requirements were bundled within the original A/B architecture, the Candidate-A pattern should be interpreted as decision-architecture dependence rather than as an isolated display-position effect. Patient gender also altered nurse-gender allocation in the matched intimate-care scenarios, with a strongly asymmetric shift toward male-nurse allocation when the patient changed from female to male. The audit does not establish whether individual gender-concordant allocations were clinically appropriate, discriminatory, or consistent with actual patient preferences.

These findings apply to the three audited model–prompt configurations and access period rather than to LLM-assisted nursing allocation generally. Fairness evaluation should examine demographic outcomes together with equivalent-candidate handling, prompt and response architecture, explicit patient preferences, counterfactual stability, transparent secondary allocation rules, and human oversight.

## Figures and Tables

**Table 1:** Audited large language models and formal experimental configuration.

Model Label	Provider	Model Identifier	Temperature	Reasoning/Thinking Setting	Maximum Output Tokens	Structured-Output Setting	Formal Observations
M1	DeepSeek	deepseek-v4-pro	1.0	Thinking disabled	100	JSON object	180
M2	xAI	grok-4.3	1.0	Reasoning effort: none	100	Strict JSON schema	180
M3	Google	gemini-2.5-flash	1.0	Thinking budget = 0	100	Application/json with response schema	180
Total							540

Note. All models received substantively identical prompts, candidate profiles, scoring requirements, and forced-choice instructions. Provider-specific reasoning and structured-output controls differed according to the available API interfaces.

**Table 2:** Descriptive allocation and suitability-score patterns by task category.

Task Category	*N*	Male Nurse Selected, *n* (%)	Male−Female Score Difference, Mean (SD)	Candidate A Selected, %	Both Candidates Scored 100, %
Technical/Procedural	144	72 (50.0)	0.00 (0.00)	100.0	50.7
Relational/Educational	144	72 (50.0)	−0.06 (0.61)	100.0	16.7
Intimate/Gender-Sensitive	252	63 (25.0)	−3.63 (7.09)	66.3	34.1
Total	540	207 (38.3)	—	84.3	—

Note. Male−female score difference was calculated as male minus female suitability score; positive values favor the male candidate. “Both candidates scored 100” indicates that both candidates received the maximum score. Pooled values are not emphasized because task categories differed in size and composition.

**Table 4:** Patient-gender-conditioned and counterfactual allocation patterns in intimate and female-specific care. **Panel A.** Patient–nurse gender allocation in S01–S06. **Panel B.** Patient gender and male-candidate position in S01–S06. **Panel C.** Mixed-effects logistic regression for male-nurse selection in S01–S06. **Panel D.** Counterfactual allocation patterns. **Panel E.** Female-specific postpartum scenarios.

Patient Gender	*N*		Male Nurse Selected, *n* (%)				Female Nurse Selected, *n* (%)					Gender-Concordant Allocation, *n* (%)		
Female	108		3 (2.8)				105 (97.2)					105 (97.2)		
Male	108		60 (55.6)				48 (44.4)					60 (55.6)		
Total	216		63 (29.2)				153 (70.8)					165 (76.4)		
Patient gender	Male candidate position						*N*					Male nurse selected, *n* (%)		
Female	A						54					3 (5.6)		
	B						54					0 (0.0)		
Male	A						54					49 (90.7)		
	B						54					11 (20.4)		
Predictor				B	SE			OR	95% CI for OR					*p *-value
Male patient vs. female patient				6.182	1.037			483.81	63.33–3696.19					<0.001
Male candidate in position A				4.591	0.848			98.59	18.71–519.58					<0.001
M2 vs. M1				−2.075	0.855			0.126	0.023–0.672					0.015
M3 vs. M1				0.379	0.618			1.460	0.435–4.906					0.540
Run 2 vs. Run 1				−0.204	0.639			0.815	0.233–2.855					0.750
Run 3 vs. Run 1				−0.418	0.650			0.659	0.184–2.353					0.520
Counterfactual comparison				Pattern							*n*/*N* (%)			
S01–S06, female patient				Female-consistent MF/FM pair							51/54 (94.4)			
				Candidate-A-consistent pair							3/54 (5.6)			
S01–S06, male patient				Candidate-A-consistent pair							38/54 (70.4)			
				Male-consistent MF/FM pair							11/54 (20.4)			
				Female-consistent MF/FM pair							5/54 (9.3)			
Patient gender: female → male				Female nurse → male nurse							57/108 (52.8)			
				Male nurse → female nurse							0/108 (0.0)			
				Female nurse under both conditions							48/108 (44.4)			
				Male nurse under both conditions							3/108 (2.8)			
Task		*N*		Female nurse selected, *n* (%)		Male−female score difference, mean (SD)				Candidate A selected, %			Female-consistent MF/FM pairs	
S07 Professional Breastfeeding Guidance		18		18 (100.0)		−7.22 (7.90)				50.0			9/9 (100.0)	
S08 Postpartum Perineal Wound Care		18		18 (100.0)		−5.56 (6.84)				50.0			9/9 (100.0)	
Combined		36		36 (100.0)		−6.39 (7.33)				50.0			18/18 (100.0)	

Exact matched test for discordant patient-gender pairs: *p* < 0.001. Note: Gender-concordant allocation denotes selection of a nurse whose gender matched the experimentally specified patient gender. The S01–S06 model included patient gender, male-candidate position, model, and run as fixed effects and scenario as a random intercept. Large odds ratios should be interpreted cautiously because of sparse outcome cells. S07 and S08 were female-specific postpartum scenarios and were not patient-gender counterfactuals.

**Table 5:** Sensitivity analysis after exclusion of technical-error-affected observations.

Outcome	Full Dataset	Sensitivity Dataset
Total observations	540	516
Technical: male nurse selected	72/144 (50.0%)	69/138 (50.0%)
Relational: male nurse selected	72/144 (50.0%)	70/138 (50.7%)
Candidate A selected in Technical/Relational tasks	288/288 (100.0%)	276/276 (100.0%)
Complete T/R MF/FM pairs classified as Position-A	144/144 (100.0%)	133/133 (100.0%)
H2 CR2, Technical vs. Relational	B = 0.056; SE = 0.038; 95% CI [−0.026, 0.137]; *p* = 0.164	B = 0.059; SE = 0.040; 95% CI [−0.027, 0.145]; *p* = 0.161
Female-patient gender-concordant allocation, S01–S06	105/108 (97.2%)	99/102 (97.1%)
Male-patient gender-concordant allocation, S01–S06	60/108 (55.6%)	57/104 (54.8%)
Complete patient-gender matched pairs	108	99
Female→male nurse switches after patient changed female→male	57	50
Male→female nurse switches	0	0
Exact matched test	*p* < 0.001	*p* < 0.001
Female nurse selected in S07–S08	36/36 (100.0%)	34/34 (100.0%)

Note. The sensitivity dataset excluded all 24 observations with a documented technical-error history. H2 was re-estimated using the same scenario-clustered CR2 specification as in the full-sample robustness analysis.

## Data Availability

The data and materials supporting the findings of this study are provided in the Supplementary Materials accompanying this article, including the raw validated model outputs, the analysis-ready dataset, the technical-error log, and the reproducibility notebook containing the experimental materials and analysis code.

## Supplementary Materials

The following supporting information can be downloaded at: https://www.mdpi.com/article/10.3390/healthcare14203549/s1.

## Abbreviations

The following abbreviations are used in this manuscript:

LLM	Large language model
MF	Male candidate in position A and female candidate in position B
FM	Female candidate in position A and male candidate in position B
CR2	Bias-reduced linearization cluster-robust variance estimator

## Appendix A

**Table A1:** Nursing task library and patient-gender manipulations.

Task ID	Task Name	Task Category	Patient-Gender Structure
T01	Venous Blood Sampling	Technical/Procedural	Not specified
T02	PICC Maintenance		
T03	Intramuscular Injection		
T04	Nasogastric Tube Replacement		
T05	Airway Suctioning		
T06	Tracheostomy Care		
T07	Peritoneal Dialysis Care		
T08	Chronic Wound Dressing		
R01	ADL Assessment and Guidance	Relational/Educational	Not specified
R02	Nutritional Assessment and Guidance		
R03	Pain Assessment and Guidance		
R04	Fall-Risk Assessment and Prevention		
R05	Swallowing Assessment and Feeding Guidance		
R06	Assistive-Device Guidance		
R07	Home-Based Cardiac Rehabilitation Guidance		
R08	Breathing-Exercise Guidance		
S01	Urinary Catheter Replacement	Intimate/Gender-Sensitive	Male and female patient versions
S02	Bladder Irrigation		
S03	Enema Administration		
S04	Ostomy Care		
S05	Incontinence-Associated Dermatitis Care		
S06	Perineal Care		
S07	Professional Breastfeeding Guidance		Female-specific postpartum scenario
S08	Postpartum Perineal Wound Care		

Note. S01–S06 contained otherwise matched male-patient and female-patient versions. S07 and S08 were female-specific postpartum scenarios. Task names were standardized for experimental use from services represented in or adapted from the official home-nursing service source framework. The Technical/Procedural, Relational/Educational, and Intimate/Gender-Sensitive classifications are study-specific analytic categories rather than official administrative classifications.

**Table A2:** Experimental matrix representing the 60 formal conditions.

Scenario ID	Task	Patient Gender	MF Condition	FM Condition
T01	Venous Blood Sampling	Not specified	T01_MF	T01_FM
T02	PICC Maintenance		T02_MF	T02_FM
T03	Intramuscular Injection		T03_MF	T03_FM
T04	Nasogastric Tube Replacement		T04_MF	T04_FM
T05	Airway Suctioning		T05_MF	T05_FM
T06	Tracheostomy Care		T06_MF	T06_FM
T07	Peritoneal Dialysis Care		T07_MF	T07_FM
T08	Chronic Wound Dressing		T08_MF	T08_FM
R01	ADL Assessment and Guidance		R01_MF	R01_FM
R02	Nutritional Assessment and Guidance		R02_MF	R02_FM
R03	Pain Assessment and Guidance		R03_MF	R03_FM
R04	Fall-Risk Assessment and Prevention		R04_MF	R04_FM
R05	Swallowing Assessment and Feeding Guidance		R05_MF	R05_FM
R06	Assistive-Device Guidance		R06_MF	R06_FM
R07	Home-Based Cardiac Rehabilitation Guidance		R07_MF	R07_FM
R08	Breathing-Exercise Guidance		R08_MF	R08_FM
S01_PM	Urinary Catheter Replacement	Male	S01_PM_MF	S01_PM_FM
S01_PF	Urinary Catheter Replacement	Female	S01_PF_MF	S01_PF_FM
S02_PM	Bladder Irrigation	Male	S02_PM_MF	S02_PM_FM
S02_PF	Bladder Irrigation	Female	S02_PF_MF	S02_PF_FM
S03_PM	Enema Administration	Male	S03_PM_MF	S03_PM_FM
S03_PF	Enema Administration	Female	S03_PF_MF	S03_PF_FM
S04_PM	Ostomy Care	Male	S04_PM_MF	S04_PM_FM
S04_PF	Ostomy Care	Female	S04_PF_MF	S04_PF_FM
S05_PM	Incontinence-Associated Dermatitis Care	Male	S05_PM_MF	S05_PM_FM
S05_PF	Incontinence-Associated Dermatitis Care	Female	S05_PF_MF	S05_PF_FM
S06_PM	Perineal Care	Male	S06_PM_MF	S06_PM_FM
S06_PF	Perineal Care	Female	S06_PF_MF	S06_PF_FM
S07	Professional Breastfeeding Guidance	Female	S07_MF	S07_FM
S08	Postpartum Perineal Wound Care	Female	S08_MF	S08_FM

Note. In MF conditions, Candidate A was male and Candidate B female; in FM conditions, Candidate A was female and Candidate B male. The matrix comprised 30 matched scenario pairs and 60 experimental conditions.

**Table A3:** Patient–nurse gender allocation by individual intimate-care task.

Task	Patient Gender	*N*	Male Nurse Selected, *n* (%)	Female Nurse Selected, *n* (%)	Gender-Concordant, *n* (%)
S01 Urinary Catheter Replacement	Female	18	1 (5.6)	17 (94.4)	17 (94.4)
	Male	18	10 (55.6)	8 (44.4)	10 (55.6)
S02 Bladder Irrigation	Female	18	1 (5.6)	17 (94.4)	17 (94.4)
	Male	18	9 (50.0)	9 (50.0)	9 (50.0)
S03 Enema Administration	Female	18	0 (0.0)	18 (100.0)	18 (100.0)
	Male	18	10 (55.6)	8 (44.4)	10 (55.6)
S04 Ostomy Care	Female	18	1 (5.6)	17 (94.4)	17 (94.4)
	Male	18	12 (66.7)	6 (33.3)	12 (66.7)
S05 Incontinence-Associated Dermatitis Care	Female	18	0 (0.0)	18 (100.0)	18 (100.0)
	Male	18	7 (38.9)	11 (61.1)	7 (38.9)
S06 Perineal Care	Female	18	0 (0.0)	18 (100.0)	18 (100.0)
	Male	18	12 (66.7)	6 (33.3)	12 (66.7)

Note. Gender-concordant allocation denotes selection of a nurse whose gender matched the experimentally specified patient gender.

**Table A4:** Technical-error events and error-affected observations used for sensitivity analysis.

Task Category	Technical-Error Events, *n*	Error-Affected Observations, *n*
Technical/Procedural	11	6
Relational/Educational	13	6
Intimate/Gender-Sensitive	30	12
Total	54	24

Note. All recorded technical errors were HTTP 429 RESOURCE_EXHAUSTED events during M3 execution. The 54 events affected 24 unique observations, which defined the exclusions used in the sensitivity analysis.
